# Impact of rifampicin-inhibitable transport on the liver distribution and tissue kinetics of erlotinib assessed with PET imaging in rats

**DOI:** 10.1186/s13550-018-0434-0

**Published:** 2018-08-16

**Authors:** Dorra Amor, Sébastien Goutal, Solène Marie, Fabien Caillé, Martin Bauer, Oliver Langer, Sylvain Auvity, Nicolas Tournier

**Affiliations:** 1 Imagerie Moléculaire In Vivo, IMIV, CEA, Inserm, CNRS, Univ. Paris-Sud, Université Paris Saclay, CEA-SHFJ, F-91400 Orsay, France; 2 Department of Clinical Pharmacology, Medical University of Vienna, Vienna, Austria; 3 Division of Nuclear Medicine, Department of Biomedical Imaging and Image-guided Therapy, Medical University of Vienna, Vienna, Austria; 4 Biomedical Systems, Center for Health & Bioresources, AIT Austrian Institute of Technology GmbH, Seibersdorf, Austria; 5 CEA, DRF, JOLIOT, Service Hospitalier Frédéric Joliot, F-91401 Orsay, France

**Keywords:** Erlotinib, Solute carrier transporter, Solute carrier O, Organic anion-transporting polypeptide, Liver, Positron emission tomography, SLCO2B1, SLCO

## Abstract

**Background:**

Erlotinib is an epidermal growth factor receptor (EGFR)-targeting tyrosine kinase inhibitor approved for treatment of non-small cell lung cancer. The wide inter-individual pharmacokinetic (PK) variability of erlotinib may impact treatment outcome and/or toxicity. Recent in vivo studies reported a nonlinear uptake transport of erlotinib into the liver, suggesting carrier-mediated system(s) to mediate its hepatobiliary clearance. Erlotinib has been identified in vitro as a substrate of organic anion-transporting polypeptide (OATP) transporters which expression does not restrict to hepatocytes and may impact the tissue uptake of erlotinib in vivo.

**Results:**

The impact of rifampicin (40 mg/kg), a potent OATP inhibitor, on the liver uptake and exposure to tissues of ^11^C-erlotinib was investigated in rats (4 animals per group) using positron emission tomography (PET) imaging. Tissue pharmacokinetics (PK) and corresponding exposure (area under the curve, AUC) were assessed in the liver, kidney cortex, abdominal aorta (blood pool) and the lungs. The plasma PK of parent ^11^C-erlotinib was also measured using arterial blood sampling to estimate the transfer rate constant (*k*_uptake_) of ^11^C-erlotinib from plasma into different tissues.

PET images unveiled the predominant distribution of ^11^C-erlotinib-associated radioactivity to the liver, which gradually moved to the intestine, thus highlighting hepatobiliary clearance. ^11^C-erlotinib also accumulated in the kidney cortex. Rifampicin did not impact AUC_aorta_ but reduced *k*_uptake, liver_ (*p* < 0.001), causing a significant 27.3% decrease in liver exposure (*p* < 0.001). Moreover, a significant decrease in *k*_uptake, kidney_ with a concomitant decrease in AUC_kidney_ (− 30.4%, *p* < 0.001) were observed. Rifampicin neither affected *k*_uptake, lung_ nor AUC_lung_.

**Conclusions:**

Our results suggest that ^11^C-erlotinib is an in vivo substrate of rOATP transporters expressed in the liver and possibly of rifampicin-inhibitable transporter(s) in the kidneys. Decreased ^11^C-erlotinib uptake by elimination organs did not translate into changes in systemic exposure and exposure to the lungs, which are a target tissue for erlotinib therapy.

## Background

Erlotinib is a reversible inhibitor of epidermal growth factor receptor (EGFR) tyrosine kinase activity, and it is approved for treatment of advanced and metastatic non-small cell lung cancer (NSCLC) with mutated EGFR [1]. However, nearly 30% of patients who are carriers of the somatic EGFR mutation do not respond adequately to therapy with tyrosine kinase inhibitors [2]. NSCLC patients, receiving the therapeutic dose of 150 mg erlotinib once daily, showed a marked interpatient variability in trough plasma concentrations and area under the curve (AUC) of 64% and 51%, respectively [3]. Variability in the pharmacokinetics (PK) of erlotinib is therefore assumed to contribute to variability in therapeutic response [4]. Furthermore, a correlation of erlotinib plasma PK parameters and treatment efficacy and occurrence of side effects was found [5, 6].

The molecular determinants for this PK variability are not fully understood. ATP-binding cassette (ABC) transporters, such as P-glycoprotein (P-gp, *ABCB1*) and breast cancer resistance protein (BCRP, *ABCG2*), were shown to play pivotal roles in restricting the intestinal absorption of erlotinib [7]. Erlotinib is mainly eliminated through hepatobiliary clearance and undergoes hepatic metabolism by cytochrome P450 (CYP) 3A4/3A5 and, to a lesser extent, by CYP1A1/2 [8]. Thus, CYP3A4 inducers and inhibitors are likely to affect erlotinib PK and may be responsible for drug-drug interactions (DDIs) [9, 10]. In addition to metabolism considerations, erlotinib is also a substrate of several transporters of importance for PK [11]. P-gp and BCRP are now recognized as major determinants for tissue distribution and clearance erlotinib [7, 12].

Recent positron emission tomography (PET) studies using carbon-11-labeled erlotinib provided novel information regarding the clearance of this drug. In baboons, injection of a high dose of unlabeled erlotinib decreased the plasma clearance of ^11^C-erlotinib, pointing to non-linear PK [13]. Similarly, a high dose of erlotinib in mice was associated with a decreased liver uptake of ^11^C-erlotinib, suggesting a saturable and possibly carrier-mediated transport mechanism for erlotinib at the basolateral membrane of hepatocytes [14]. This observation was recently confirmed in healthy human volunteers using a similar saturation approach [15].

The hepatic uptake of various xenobiotics is mediated by sinusoidal membrane transporters, mainly solute carrier (SLC) transporters, as a prerequisite for hepatobiliary elimination [16]. Three members of the organic anion-transporting polypeptide (OATP) family (*SLCO* gene), namely OATP1B1 (*SLCO1B1*), OATP1B3 (*SLCO1B3*), and OATP2B1 (*SLCO2B1*), are expressed in hepatocytes and are considered of importance for PK [11]. Recent in vitro studies demonstrated that erlotinib is selectively transported by OATP2B1 among the other OATP transporters expressed in hepatocytes [15]. OATP2B1 expression is not restricted to the liver and may also play a role in the uptake of its substrates into other organs [17]. OATP2B1 function may thus account for variability in the distribution of erlotinib to the liver and to target/vulnerable tissues, with consequences for treatment efficacy and safety. OATP function was thus hypothesized to control the liver uptake of erlotinib with consequences for plasma and tissue kinetics in vivo.

In the present study, ^11^C-erlotinib PET imaging was performed in anesthetized rats in the absence and the presence of the broad spectrum OATP inhibitor rifampicin to understand whether rifampicin-inhibitable uptake transport may be responsible for the pharmacokinetic variability of erlotinib in patients.

## Methods

### Animals

Fourteen male Sprague-Dawley rats (Janvier, France) were included in the study (392 ± 31 g in weight at the time of experiment). Animals were housed in a temperature and humidity-controlled room with a 12-h light/dark cycle and with access to food and water ad libitum. Animal studies were conducted in accordance with the French legislation and European directives on the use of animals in research. The protocol has been accepted by a local ethics committee for animal use (protocol number A16/057).

### Chemicals and radiochemicals

6-*O*-desmethyl erlotinib was purchased from Syncom (The Netherlands). Rifampicin (Rifadine^®^) was purchased from Sanofi-Aventis (France). ^11^C-erlotinib was synthesized by ^11^C-methylation of 6-O-desmethyl erlotinib as previously described [18]. The radiochemical purity of ^11^C-erlotinib was greater than 98% with a molar activity of 152 ± 65 GBq/μmol (decay-corrected to the end of bombardment).

### PET imaging

PET imaging was performed using an Inveon^®^ microPET system (Siemens, Germany). Anesthesia was induced and further maintained for the whole scan duration using 4% and 2% of isoflurane in O_2_, respectively. A tail vein was catheterized for radiotracer injection, and rats were then positioned on a heated bed with their abdominal region located at the center of the field of view. Dynamic PET acquisition (60 min) started at the time of ^11^C-erlotinib injection (injected amount 33 ± 7 MBq). ^11^C-erlotinib dynamic PET data were sorted into 24 frames with time durations of 3 × 0.5 min, 6 × 1 min, 5 × 2 min, 4 × 3 min, 2 × 4 min, 4 × 5 min, and 1 × 2.5 min. Dynamic images were reconstructed using the FORE+OSEM2D algorithm including normalization, attenuation, scatter and random corrections.

PET experiments were performed in the absence (control group, *n* = 4 animals) and in the presence (rifampicin group, *n* = 4 animals) of an intravenous (i.v.) pretreatment with rifampicin (40 mg/kg), given as a bolus at 5 min before radiotracer injection. Acute rifampicin administration was previously shown to inhibit OATP function in rat using different OATP-substrate probes [19–22].

### Data analysis

PET data analysis was performed using PMOD software (version 3.8; PMOD Technologies, Zurich, Switzerland). For each animal, volumes of interest (VOIs) were manually drawn for the abdominal aorta (clearly observed at early time frames), liver, lungs, and kidney cortex. Corresponding time-activity curves (TACs) were generated. Radioactivity was corrected for injected dose and animal weight, and data were expressed in units of standardized uptake value (SUV). The highest concentration value of the time-activity curves was defined as SUV_max_. Area under the TACs was estimated from the start to the end of acquisition for the abdominal aorta, the liver, the lungs, and the kidneys (AUC_aorta_, AUC_liver_, AUC_lungs_, and AUC_kidneys_; SUV.min) using GraphPad Prism software (CA, USA).

### Arterial input function

In parallel conditions and for each group, one additional animal was used to measure a metabolite-corrected arterial input function of ^11^C-erlotinib, obtained from arterial blood sampling, gamma counting, and HPLC analysis (injected amount 58 and 46 MBq for control and rifampicin condition, respectively). Twenty blood samples (50 μL) were collected at selected times from the femoral artery to describe the time course of total radioactivity in arterial plasma after radiotracer injection. Samples were centrifuged (3 min, 2054×*g*, 4 °C), and radioactivity in cell-free plasma (25 μL) was determined using a Cobra Quantum^®^ (D5003, Perkin-Elmer) gamma counter. Additional samples (500 μL) were withdrawn at 5, 10, 15, 30, and 60 min after injection to determine the parent fraction of unmetabolized ^11^C-erlotinib in plasma using radio-HPLC analysis as previously described [13]. For each condition, the parent fraction of ^11^C-erlotinib versus time was fitted using a 1-exponential decay equation and applied to the total radioactivity kinetics to estimate a representative metabolite-corrected arterial input function of ^11^C-erlotinib. AUC_plasma_ expressed as SUV.min represents the AUC of the metabolite-corrected arterial input function, obtained using arterial blood sampling from 0 to 60 min after radiotracer injection.

### Determination of parent ^11^C-erlotinib in the liver

In parallel to the PET imaging experiments, the parent fraction of ^11^C-erlotinib was determined in the liver at 15 min after radiotracer injection. Two additional rats per condition received a ^11^C-erlotinib injection (injected amount 43 and 40 MBq for the control condition; 32 and 19 MBq for the rifampicin condition) under isoflurane anesthesia. After 15 min, animals were decapitated and liver fragments were removed and sonicated for extraction using acetonitrile. After centrifugation, the supernatant was counted for radioactivity and subjected to radio-HPLC analysis.

### Rate constant for the transfer of ^11^C-erlotinib from blood to tissues

Rate constants for the transfer of ^11^C-erlotinib from plasma to the liver, kidneys, and lungs (*k*_uptake, liver_, *k*_uptake, kidney_ and *k*_uptake, lung_) were calculated using a previously described graphical method (integration plot analysis) and the following equation [21]:

$$ {C}_{\mathrm{t},\mathrm{organ}}/{C}_{\mathrm{t},\mathrm{plasma}}={k}_{\mathrm{uptake},\mathrm{organ}}\times \left({\mathrm{AUC}}_{0-\mathrm{t},\mathrm{plasma}}/{C}_{\mathrm{t},\mathrm{plasma}}\right)+{V}_{\mathrm{E}}, $$

where *C*_t, organ_ is the concentration of radioactivity in the investigated organ at time *t*, determined with PET. *C*_t, plasma_ is the concentration of parent ^11^C-erlotinib in the blood at time *t*, determined by arterial blood sampling, and AUC_0 − t, plasma_ is the area under the blood concentration-time curve of ^11^C-erlotinib from time 0 to time *t*. *k*_uptake_ is equal to the slope value of the regression line obtained by performing linear regression analysis of a plot of *C*_t, organ_/*C*_t, plasma_ versus AUC_0 − t, plasma_/*C*_t, plasma_. *V*_E_ is the *y*-intercept of the integration plot, representing the initial distribution volume of ^11^C-erlotinib in the organ. Using this approach, linear uptake was observed from 0.25 to 6 min after tracer injection for the liver and from 1.25 to 6 min for the kidneys and lungs.

### Statistical analysis

Data are reported as mean ± SD. Statistical analysis was performed using a two-way ANOVA with “treatment” and “organ” as factors. Statistical significance was set at *p* < 0.05.

## Results

**Fig. 1:**
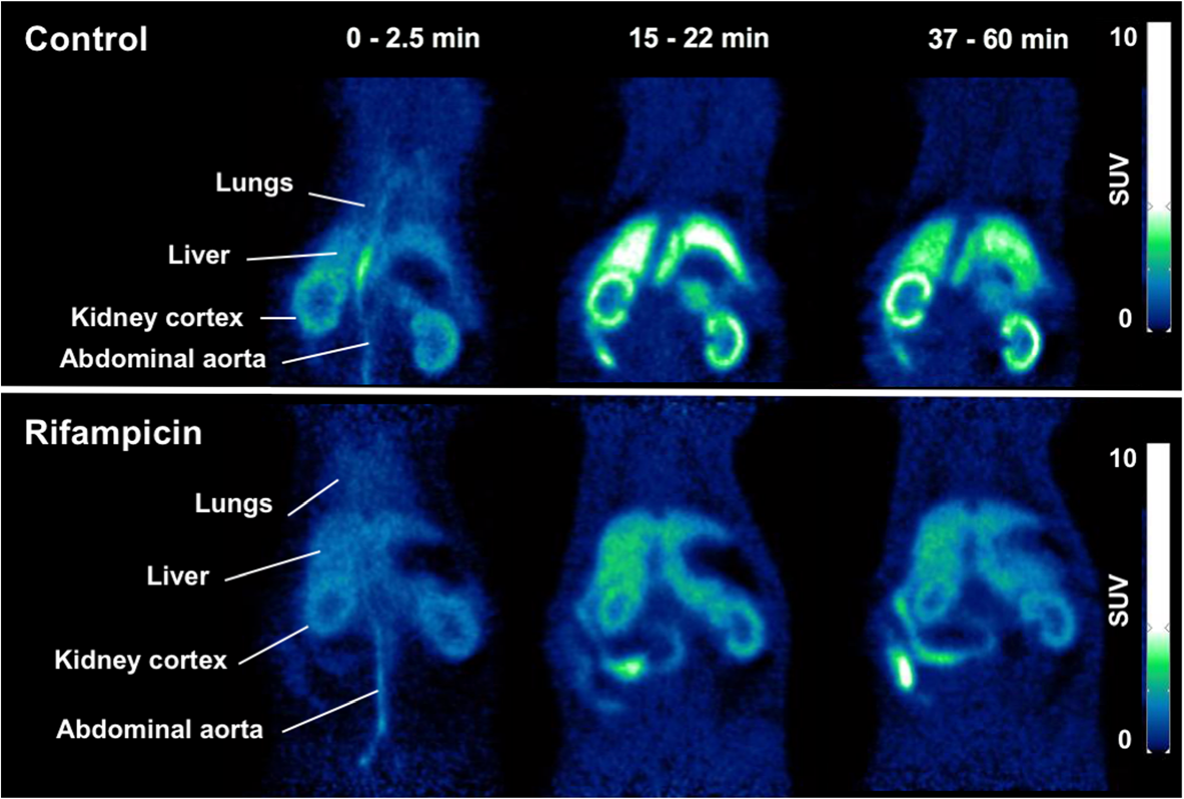
Representative PET images of the rat abdominal region after ^11^C-erlotinib injection in the control condition and after rifampicin pretreatment (40 mg/kg i.v; 5 min before ^11^C-erlotinib injection)

Figure 1 shows representative PET images centered on the abdominal region for a control and a rifampicin-pretreated rat. Radioactivity was primarily localized in the liver within the first minutes after ^11^C-erlotinib injection, and then it gradually moved to the intestine, thus dynamically highlighting the predominantly hepatobiliary elimination of radioactivity (Fig. 1). Radioactivity also accumulated in the kidneys, which were best visualized on late summation PET images. The lungs were visible in the very first frames only, probably due to their high vascular content (Fig. 1).

**Fig. 2:**
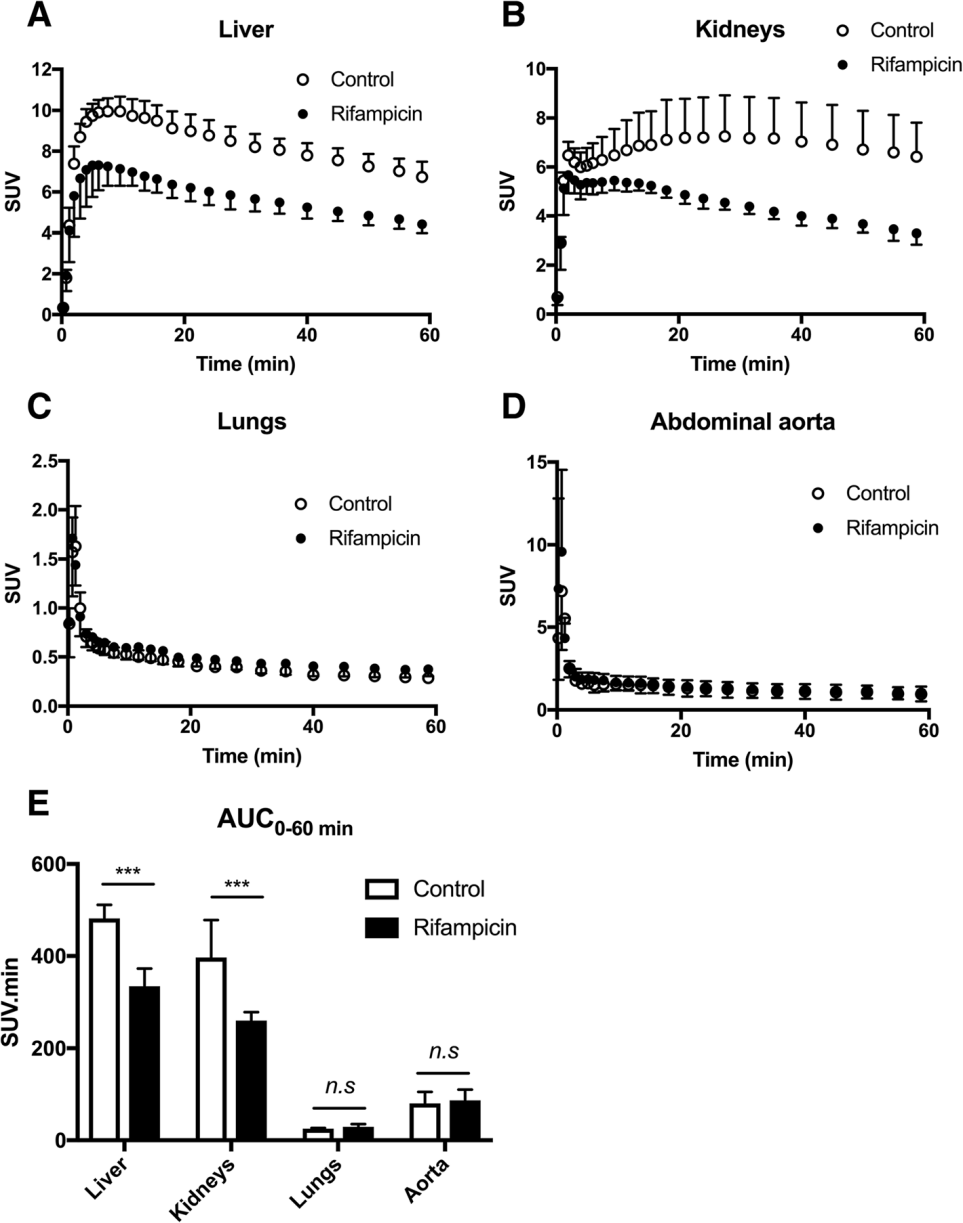
Time-activity curves of ^11^C-erlotinib in the liver (**a**), kidneys (**b**), and lungs (**c**) and abdominal aorta (**d**) in the absence (control) and the presence of OATP inhibition with rifampicin (40 mg/kg i.v.). Corresponding exposure to tissues, expressed as area under the curve (AUC) in each organ, is reported in (**e**). Data are mean (*n* = 4) ± 1. S.D. ***, *p* < 0.001, *n.s.*, non-significant

From time points > 3 min after injection, hepatic accumulation of radioactivity was lower in rifampicin-pretreated rats than in control animals (Fig. 1). SUV_max_ in the liver was significantly lower in rifampicin-treated animals (7.3 ± 1.2) as compared with control animals (9.9 ± 0.7, *p* < 0.05). Rifampicin pretreatment caused a significant ~ 27% decrease in AUC_liver_ (*p* < 0.001; Fig. 2). In animals sacrificed at 15 min after injection, the percentage of unmetabolized ^11^C-erlotinib in the liver ranged from 96.4 to 100% in the control group and from 96.0 to 100% in the rifampicin-treated group, respectively. This shows a limited contribution of radiolabeled metabolites to the PET signal in the liver within the first 15 min of scanning. AUC_liver_ from 0 to 15 min was significantly lower in rifampicin-treated animals (103.6 ± 15.2 SUV.min) as compared with the control animals (129.4 ± 10.1 SUV.min, *p* < 0.05).

Radioactivity concentrations in the kidney cortex were also lower in the rifampicin-treated group, and the difference tended to increase with time (Fig. 2). AUC_kidney_ was markedly reduced in rifampicin-treated animals (*p* < 0.001). The lungs showed a much lower exposure to radioactivity compared to the liver and the kidneys (Fig. 2). No significant differences in AUC_lung_ were observed between the two groups (Fig. 2).

**Fig. 3:**
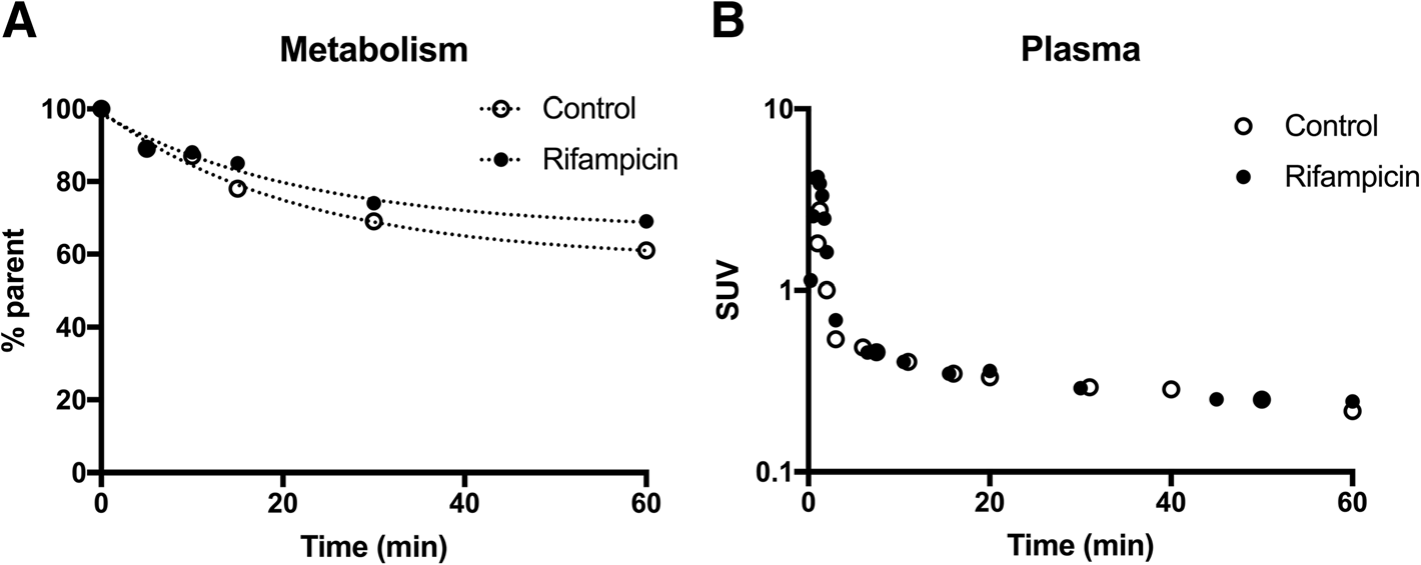
Metabolism and plasma kinetics of ^11^C-erlotinib in the absence (control) and the presence of rifampicin (40 mg/kg i.v.). Data were obtained using arterial blood sampling (femoral artery) in additional rats (1 animal for each condition). In **a**, the percentage of parent (unmetabolized) ^11^C-erlotinib versus time is displayed. Corresponding plasma kinetics of parent ^11^C-erlotinib (metabolite-corrected arterial input function) are shown in **b**

Unexpectedly, rifampicin did not impact the image-derived blood kinetics. AUC_aorta_ (80.4 ± 24.7 SUV.min) was not increased by rifampicin treatment (86.6 ± 23.8 SUV.min; *p* > 0.05). Radiotracer metabolism and binding to blood cells in either the presence or the absence of rifampicin may interfere with the estimation of the transfer of parent ^11^C-erlotinib from plasma to tissues. The plasma kinetics of parent ^11^C-erlotinib was therefore measured in one animal of each condition, from repeated arterial blood sampling, in order to provide a representative metabolite-corrected arterial input function and calculate *k*_uptake_ in tissues. The percentage of parent ^11^C-erlotinib versus time was similar in the control and the rifampicin-pretreated rat (Fig. 3a). At 1 h after radiotracer injection, the percentage of unmetabolized ^11^C-erlotinib was 61% and 69% without and with rifampicin pretreatment, respectively. ^11^C-erlotinib plasma concentrations peaked rapidly after i.v. injection, followed by a rapid wash out (Fig. 3b). A plateau was reached after 15 min (SUV_15 min_ = 0.34) with SUV_60 min_ = 0.22 and 0.24 for the baseline and the rifampicin pretreated rats, respectively.

**Fig. 4:**
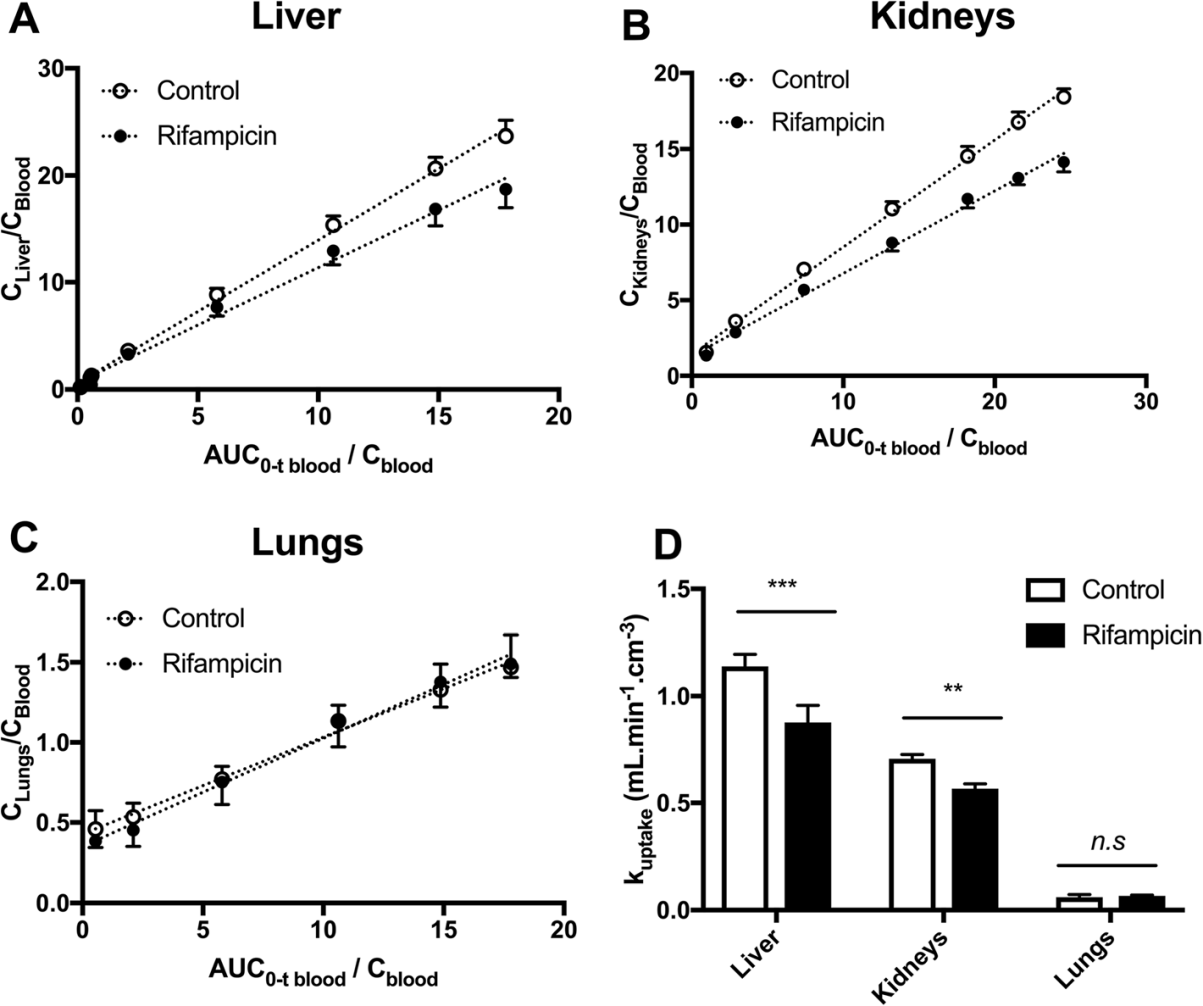
Estimation of the rate constant for the transfer of ^11^C-erlotinib from plasma into tissues. Integration plots of ^11^C-erlotinib uptake by the liver (**a**), kidneys (**b**), and lungs (**c**) are shown. *K*_uptake_ values are reported in **d**. Data are mean (*n* = 4) ± 1. S.D. *** *p* < 0.001; ** *p* < 0.01; *n.s* non-significant

The lower liver and kidney exposures to radioactivity observed in the rifampicin-pretreated animals were consistent with significant decreases in *k*_uptake, liver_ and *k*_uptake, kidney_ (Fig. 4). There was no effect of rifampicin on *k*_uptake, lung_, which was consistent with the absence of an effect of rifampicin on AUC_lung_ (Fig. 2).

## Discussion

PET imaging is an appealing strategy to study the impact of OATP transporters on the tissue distribution of radiolabeled drugs [23]. In vitro studies using transfected cells showed that erlotinib is specifically transported by the human OATP2B1 [15]. Recent PET studies have demonstrated saturable liver uptake of ^11^C-erlotinib in humans [15]. To the best of our knowledge, the transport of erlotinib by the rodent rOATP2B1 has not been addressed but a similar saturation of the liver uptake of ^11^C-erlotinib was also observed in mice [14]. We therefore hypothesized that OATP inhibition with rifampicin may decrease the liver uptake of ^11^C-erlotinib with consequences for drug exposure to the lungs, as the therapeutic target tissue of erlotinib.

OATP transporters are recognized as the key determinants for the PK of many drugs [11]. They are expressed in the sinusoidal membrane of hepatocytes and were shown to control the uptake of their substrates from the blood into the liver. Conventional PK studies, based on the determination of drug concentrations in the plasma, have shown how alterations in hepatic OATP1B1 and OATP1B3 function may influence the plasma PK of diverse drugs. Genetic polymorphisms of the *SLCO* genes [24] as well as DDIs involving inhibitors of OATP transporters [25] have been deemed of clinical importance for PK variability. The importance of OATP2B1 in the liver is less understood, probably due to its overlapping substrate specificity with OATP1B1 and OATP1B3 [25]. Expression of OATP2B1 has been detected at many other blood-tissue interfaces than the sinusoidal membrane of hepatocytes, suggesting that OATP2B1 may control the target/vulnerable tissue exposure to its substrates in addition to its impact on hepatobiliary clearance [17].

Rifampicin is a prototypical OATP inhibitor used in clinical studies in drug development to assess the PK importance of OATP function [11, 26]. In vitro, rifampicin is a potent inhibitor of the human OATP1B1 (IC_50_ = 1.9 ± 0.16 μM) and OATP1B3 (IC_50_ = 6.4 ± 0.5 μM) and a weaker inhibitor of OATP2B1 (IC_50_ = 91.0 ± 14.6 μM) [27, 28]. Rifampicin was also shown to inhibit several rat OATPs including the liver-specific rOATP1B2 (*K*_i_ = 0.79 μM). Rifampicin also inhibits rOATP1A1 (*K*_i_ = 18 μM) and rOATP1A4 (*K*_i_ = 1.4–2.9 μM) [29–31]. The inhibition potency of rifampicin for other SLC transporters of PK importance has been studied in vitro and suggests a relatively good specificity for OATP transporters. Indeed, rifampicin inhibited neither organic cation transporter 1 (OCT1, *SLC22A1*, *K*_i_ > 100 μM) nor organic anion transporter 2 (OAT2, *SLC22A7*), which are both expressed in the sinusoidal hepatocyte membrane. Rifampicin most likely does not inhibit organic cation transporter 2 (OCT2, *SLC22A2*) and organic anion transporter 3 (OAT3, *SLC22A8*) function in vivo [32]. Plasma concentrations of rifampicin, when administered to rats at a dose of 40 mg/kg i.v., ranged from 100 to 300 μM, which exceeded the reported in vitro *K*_i_ values for inhibition of OATP transporters [20, 22]. The selected rifampicin dose was previously shown to inhibit the rOATP-mediated liver uptake of ^11^C-dehydropravastatin and ^11^C-rosuvastatin, as assessed with PET imaging in rats [20, 22]. In our study, we chose a simple i.v. bolus administration protocol, which did not require constant infusion during PET scanning.

Our PET data showed a predominant liver accumulation of radioactivity, which was consistent with hepatobiliary elimination of erlotinib [33]. A high hepatic accumulation of ^11^C-erlotinib has also been observed in mice and in humans [14, 15]. Rifampicin pretreatment significantly decreased the liver uptake and subsequent liver exposure to ^11^C-erlotinib. This suggested a role of sinusoidal rOATP transporters in controlling the hepatobiliary elimination of erlotinib. Radioactivity in the liver, analyzed at 15 min after injection, mainly consisted of unmetabolized ^11^C-erlotinib, regardless of the presence or absence of rOATP inhibition. This suggested that the quantification of *k*_uptake, liver_ values was not influenced by radiotracer metabolism and accurately reflected the transfer constant of parent ^11^C-erlotinib from the blood into the liver.

Rifampicin is also a potent inhibitor of MRP2 (*ABCC2*) expressed at the canalicular side of hepatocytes [19]. Erlotinib is not a substrate of MRP2 [7]. Consistently, the slopes of ^11^C-erlotinib elimination from the liver were similar in the control and rifampicin-treated animals suggesting a predominant impact of rifampicin in reducing the basaloteral uptake of ^11^C-erlotinib by hepatocytes [19].

Control ^11^C-erlotinib PET images showed a relatively high uptake of the radioactivity in the kidney cortex. Unexpectedly, rifampicin also decreased the uptake of ^11^C-erlotinib into the kidneys, which led to a significant 30.4% decrease in kidney exposure. This effect is consistent with the saturable uptake of ^11^C-erlotinib by the kidneys reported in mice [14]. A similar phenomenon has been observed for the OATP substrate ^11^C-rosuvastatin, which kidney uptake was also decreased after rifampicin administration [20]. This suggested a role for rifampicin-inhibitable transporter(s) expressed in rat kidney epithelial cells in controlling the uptake and subsequent kidney exposure to erlotinib. The decrease in the kidney exposure was related to the decrease in *k*_uptake, kidneys_. This suggests a role for a basolateral blood-to-kidney transport rather than secretion or reabsorption process at the tubular level, as previously reported for ^11^C-rosuvastatin [20]. Rifampicin is an inhibitor of OAT1 and OAT2 but not OAT3 expressed at the basolateral membrane of kidney proximal tubules [20]. However, erlotinib is neither transported by OAT1 nor OAT2 but is a substrate of OAT3 [12]. Erlotinib is also a substrate of OCT2 which is not inhibited by rifampicin [12, 27]. Rifampicin is not an inhibitor of OATP4C1 [34] which importance for erlotinib transport is not known. Previous mice studies have shown that parent unmetabolized ^11^C-erlotinib could not be detected in urines [14]. It is therefore difficult to address whether changes in the ^11^C-erlotinib uptake by the kidneys translated into changes in the urinary clearance of the tracer. Species differences in the expression and function of membrane transporters may occur between rats and humans. Clinical experiments using ^11^C-erlotinib along with urine sampling would be useful to finally address the putative impact of these rifampicin-inhibitable transporter(s) in the uptake by the kidney tissue and whether it could drive any change in the renal elimination of erlotinib and its metabolites.

The lungs are the main target tissue for erlotinib therapy in patients. A decrease in the distribution of ^11^C-erlotinib to the major clearance organs liver and kidneys was expected to lead to an increase in plasma concentrations and a concomitant increase in lung exposure. Increase in plasma concentrations has been observed for ^11^C-rosuvastatin, as a consequence of decreased hepatic and renal uptake by rifampicin [20]. Interestingly, our results indicated that rifampicin decreased the distribution of erlotinib into the main elimination organs of the body with no or negligible consequences for blood and lung exposure. The lungs contain a high vascular fraction, and the concentration of radioactivity measured with PET in the lungs can therefore be expected to be influenced by the plasma kinetics of ^11^C-erlotinib, which did not differ between the control and rifampicin-treated rats.

The lack of an effect of rifampicin on systemic exposure suggested a limited role for rifampicin-inhibitable transporter(s) in controlling the total clearance of erlotinib. First, it can be hypothesized that a larger decrease in the uptake of ^11^C-erlotinib by the liver or kidneys would be necessary to detect changes into plasma PK. Indeed, saturation of the liver and kidney transport by unlabeled erlotinib led to significant changes in the plasma PK of ^11^C-erlotinib [14]. This suggests that substantial changes into the uptake of drugs by elimination organs may not directly translate into changes in plasma PK. This phenomenon could also be explained by compensatory mechanisms, such as a shift from hepatobiliary elimination to another (probably urinary) elimination pathway, which was previously reported for ^11^C-erlotinib in mice [14, 35]. It may also be hypothesized that efficient reabsorption of ^11^C-erlotinib at the enterohepatic level, which may not be inhibited by rifampicin, may counteract the decreased transport of ^11^C-erlotinib throughout hepatocytes. The impact of transporter function on the enterohepatic circulation of drugs is difficult to assess using PET. Nevertheless, such phenomenon would probably translate into changes in the early plasma kinetics [36], which was not observed in our experiments.

Rifampicin had no significant impact on *k*_uptake, lung_, suggesting that erlotinib uptake by the lungs is independent of rOATP transporter function. This is consistent with previous studies showing that rOATP1A1, rOATP1A4, and rOATP1B2, which can be inhibited with rifampicin, are not expressed in the lungs [37].

Imaging data obtained in the present animal study should be interpreted with respect to a clinical extrapolation. First, the expression, function, and tissue distribution of OATP transporters differ between rats and humans [38]. Second, the rifampicin dose used in this study was considerably higher than the rifampicin dose used to test OATP-mediated DDIs in humans (10 mg/kg). Third, we used in the present study a microdose of erlotinib, on a relatively short time, which may not reflect the PK behavior of a pharmacological dose of erlotinib. In fact, a previous ^11^C-erlotinib PET study in healthy volunteers suggested that OATP2B1-mediated liver uptake of erlotinib was negligible at pharmacological doses, due to saturation of transport activity [15]. Nevertheless, our results demonstrate that discrepancies may exist between the PK of drugs in elimination organs, their systemic PK, and subsequent exposure to target tissues. Our data suggest that OATP-mediated DDIs, with consequences for liver and kidney exposure, may not necessarily translate into changes in systemic PK. Previous studies have demonstrated the feasibility of ^11^C-erlotinib PET imaging in healthy volunteers [15], which may be combined with rifampicin administration [39]. A clinical translation of our results is thus possible to validate ^11^C-erlotinib as a PET probe for imaging of OATP2B1 function and to assess the impact of OATP inhibition on the liver distribution and PK of erlotinib in humans.

Passive diffusion is often assumed to be the main mechanism for erlotinib to enter cancer cells and reach the EGFR kinase domains. However, it is not known whether TKIs such as erlotinib can freely cross membranes by passive diffusion or whether SLC transporters play a role in the tumor uptake [40]. Such phenomenon may control drug delivery to the tumor and account for therapeutic efficiency, with limited impact on the plasma PK. ^11^C-erlotinib PET imaging, performed with and without transporter inhibitors such as rifampicin, could be useful to address the impact of membrane transporters in controlling the uptake of erlotinib by tumor cells in vivo [41, 42].

## Conclusion

In this study, the impact of rifampicin on erlotinib uptake by the liver and kidneys was assessed using ^11^C-erlotinib PET imaging in rats. Unexpectedly, despite significant decreases in liver and kidney exposure, rifampicin did not affect systemic exposure and subsequent exposure to the lungs as the main therapeutic target tissue of erlotinib. The consequences for erlotinib PK of these newly reported rifampicin-inhibitable transports, possibly OATP, should be addressed in humans using ^11^C-erlotinib PET imaging [39].

## Abbreviations

ABC: ATP-binding cassette
AUC: Area under the curve
CYP: Cytochrome P-450
EGFR: Epidermal growth factor receptor
NSCLC: Non-small-cell lung cancer
OAT: Organic anion transporter
OATP: Organic anion-transporting polypeptide
PET: Positron emission tomography
P-gp: P-glycoprotein
PK: Pharmacokinetics
SLC: Solute carrier
SUV: Standardized uptake value
